# Abstracts from the the 7^th^ Annual Scientific Conference of The Saudi Thoracic Society and the 8^th^ Annual Asthma Meeting 17-19 March 2009 Marriott Hotel, Riyadh, Saudi Arabia

**Published:** 2009

## Survey of mycobacterium tuberculosis and other pathogenic bacteria rate in bronchial exudates of patients with pulmonary disorders

**Abbas Mirshafiey, Mohammad Hossein Salari, Robabah Hafezi**

Department of pathobiology, School of Public Health and Institute of Public Health Research, Tehran University of Medical Sciences, Tehran, Iran

**Objective:** The purpose of this study was to investigate Mycobacterium tuberculosis and other pathogenic bacteria rate in bronchial exudates of patients with pulmonary disorders. **Materials and Methods:** In this study 136 bronchial exudates and 136 blood samples of patients with pulmonary disorders were investigated by culture method and cold agglutination test and questionnaire completed for each patient. Collected bronchial exudates were cultured on selected media (pplo agar, Lowenstein Jensen, blood agar, Endo agar, and Chocolate agar) and incubated at 37°C. All isolates were characterized to species level by conventional biochemical tests. Then, collected data were analyzed by statistical package for social science (SPSS) and chi-square program. **Results:** The results show that, isolated pathogenic bacteria were Mycobacterium tuberculosis (3.3%), Mycoplasma pneumonia (9.1% positive by culture method; 2.9% positive by cold agglutination test), Streptococcus pneumonia (10.2%), Klebsiella pneumonia (15.3%), Haemophilus influenzae (10.2%), Staphylococcus aureus (8.5%) and Pseudomonas aeroginosa (52.5%). **Conclusion:** Bacterial pulmonary infections are still an important problem and elimination of this disease may not be feasible without increased efforts in prevention, diagnosis and treatment.

**Key words:** Pathogenic bacteria, patients, bronchial exudates, pulmonary disorders

## Exhaled nitric oxide in stable chronic obstructive pulmonary disease

**Mohammed F. S. Beg, Mohammad Al Zoghaibi, Abdullah A Abba^1^, Syed S. Habib**

Departments of Physiology and ^1^Medicine, King Saud University and King Khalid University Hospital, Riyadh, Saudi Arabia

**Study Objective**: The objective of the study was to test the hypothesis that fraction of exhaled nitric oxide (FENO) is elevated in nonsmoking subjects with stable chronic obstructive pulmonary disease (COPD) and compare it with the results in patients with asthma and a control population. **Design**: Cross-sectional study. **Setting and Subjects**: Pulmonology Clinic at a University Hospital. Twenty five control subjects, 25 steroid naïve asthmatics and 14 COPD patients were studied. All patients were nonsmokers and stable at the time of the study. **Measurement**: All subjects completed a questionnaire and underwent spirometry. Exhaled nitric oxide was measured online by chemiluminescence using single-breath technique. **Results:** All study subjects were males. Subjects with stable COPD had significantly higher values of FENO than controls (56.54 ± 28.01 vs 22.00 ± 6.69; *P* = 0.0001) but lower than subjects with asthma (56.54 ± 28.01 vs 84.78 ± 39.32 *P*= 0.0285). The FENO values in COPD subjects were inversely related to FEV1/FVC ratio. There was a significant overlap between the FENO values in COPD and control subjects. **Conclusion:** There is significant elevation in FENO in patients with stable COPD but the elevation is less than in asthmatic subjects. Its value in clinical practice may be limited by the significant overlap with control subjects.

## Sleep habits and academic performance in medical students

**Abdulrahman M. Alaseem, Abdulmajeed A. Alzkri, Deema Harakti, Maram Gattan, Muhammad M. Sharif, Ahmed S. BaHammam**

The University Sleep Disorders Center, College of Medicine and King Khalid University hospital, King Saud University, Riyadh, Saudi Arabia

**Introduction:** Sleep habits and disturbances have been shown to negatively affect school performance at the level of school–aged students. However, limited numbers of studies have assessed the effect of sleep habits and disturbances on the academic performance among medical students as a specific group. **Objectives:** This study was designed to assess the association between sleep patterns/habits academic performance among medical students (males and females) at different academic levels. **Materials and Methods:** Participants in this cross sectional study were healthy medical students in the first, second and third academic levels of the college of medicine, King Saud University, Riyadh, Saudi Arabia. The study was conducted during December 2008. A self administered questionnaire was distributed to students to asses age, academic level, academic performance based on the accumulative grades, sleep-wake schedule, naps, quality of sleep based on self-satisfaction with sleep, total sleep time at night and day time sleepiness using the Epworth Sleepiness Scale (ESS). Day time sleepiness was defined as an ESS score of >10. Students who didn't report their accumulative grades, have chronic illnesses or drink alcohol were excluded. **Results:** The final analysis included 410 students (273 males and 137 females) with a mean age of 20.37 ± 1.13 years and a body mass index (BMI) of 25.01 ± 8.6 kg/m^2^. One hundred and fifteen students (28%) were defined to be in group1 (those with grade A) and 295 students (72%) to be in group2 (those with grades of B, C or D). Group1 students went to bed earlier during weekdays (23.8 ± 1.70) compared to those in group2 (00.2 ± 1.5), *P*=0.018. Also, they had longer total sleep time (TST) at night during weekdays (6.28 ±1.45 hours) than students in group 2 (6.02 ±1.25), *P*=0.073. ESS score was significantly less in group1 students (6.88 ± 3.64) than group2 students (8.03 ± 3.89), *P*=0.007. Additionally, 24 students (20.9%) of group1 reported that they had chronic sleep complaints versus 101 students (34.2 %) in group 2, *P*=0.008. Sixty three students (55.3%) in group1 felt sleepy during daytime in comparison to 198 (68.8%) in group 2 students, *P*=0.01. Only one smoker (0.9%) in group1 versus 23 (7.9%) smokers in group 2, *P*=0.007. Obesity was significantly more common in group 2 students (16.3%) than group1 students (7.8%), *P*=0.026. **Conclusion:** The present study showed that lower academic performance is associated with the presence of sleep complaints, poor sleep habits, shortened total sleep time at night and daytime sleepiness.

**Key words:** Sleep habits, academic performance, medical students

## Circadian pattern of sleep, energy expenditure, body temperature and heat flux during ramadan fasting using the ArmBand

**BaHammam A, Sharif MM, Albabtain M, Alrajeh M,**

The University Sleep Disorders Center, College of Medicine, King Saud University

**Introduction:** Conflicting data has been reported regarding the effects of Ramadan fasting on the circadian pattern of sleep and other physiological parameters. Therefore, we conducted this study to assess the circadian changes in sleep, energy expenditure and body temperature during Ramadan fasting in healthy young adults. **Materials and Methods:** Six young (age 20.5 ± 2.9) non-smokers healthy adults with a body mass index of 22.6 ± 2.9 kg/m^2^ were included. Data using the ArmBand (SenseWear Pro2 Armband, BodyMedia Inc, Pittsburgh, PA, USA)was collected during the last week of Shaaban (baseline (BL) data) and during the first (R1) and second (R2) weeks of Ramadan. The ArmBand is a validated metabolic body monitoring system that will record body temperature, total energy expenditure, sleep efficiency, and duration and the circadian distribution over 24 hours. It was placed around the arm of the volunteer over the triceps during the study period. The collected data was downloaded into a PC and an advanced analysis was used to extract and analyze the needed information. **Results:** Table 1 presents the data for the three weeks. Apart from decreased energy expenditure and delayed wake up time during R1, there were no differences in other measured parameters including bedtime, total sleep time, nap time and duration and circadian pattern of body temperature and heat flux between Ramadan and BL. **Conclusion:** Ramadan fasting does not affect the circadian pattern of sleep or body temperature.

**Table 1:** Circadian pattern os sleep over 3 weeks

Variable	BL	R1	R2
TST (hr)	4.53 ± 2.3	3.8 ± 2.1	3.3 ± 2.2
Nap duration (hr)	2.7 ± 2.5	3.5 ± 2.4	4.0 ± 1.8
TST + Nap (hr)	7.2 ± 1.1	7.1 ± 0.7	7.3 ± 1.2
Nap bedtime (hr)	11.3 ± 1.3	11.4 ± 2.4	10.9 ± 2.6
Nap wake up time (hr)*	13.9 ± 1.9	14.9 ± 2.3	14.1 ± 1.1
Night bedtime (hr)	6.0 ± 4.5	4.4 ± 0.9	5.3 ± 1.1
Night rise time (hr)	8.1 ± 2.4	9.7 ± 3.3	9.1 ± 3.1
Heat flux	63.76	63.9 ± 9.4	63.9 ± 9.4
Skin temperature (°C)	33.0 ± 0.5	33.1 ± 0.5	33.1 ± 0.5
Near body temperature (°C)	32.9 ± 0.4	32.9 ± 0.4	32.9 ± 0.41
Number of steps/min	8.7 ± 3.1	7.2 ± 2.7	7.4 ± 2.6
aPhysical activity*	0.1 ± 0.05	0.1 ± 0.04	0.1 ± 0.03
Energy expenditure kcal/min*	2.2 v 0.4	2.0 ± 0.4	2.0 ± 0.3
METS*	2.0 ± 0.2	1.7 ± 0.2	1.8 ± 0.2

BL: Baseline in Shaaban; R1: first week of Ramadan; R2: second week of Ramadan

* *P*<0.5, on pair wise comparison, all the significance are due to BL and R1. No significance is observed between BL & R2 and R1 & R2.

a Physical activity is measured when METS>=3.0 and is calculated on the mean physical activity / minute for the whole duration. This definition is given by the Body Media official maker of Arm Band.

## The utility of ApneaLink™ as a screening tool for obstructive sleep apnea

**Ahmed BaHammam, Munir Chaudhery, Shaffi Shaikh**

The University Sleep Disorders Center, College of Medicine, King Saud University

**Introduction:** Sleep medicine service in Saudi Arabia is underdeveloped partly due to the unavailability of proper sleep disorders laboratories and trained personnel. Therefore, the need for simple validated portable devices to diagnose obstructive sleep apnea (OSA) becomes essential. For this reason, we designed this study to assess the validity and utility of ApneaLink ™ device (ResMed Corporation, Poway, Calif) as a portable for screening patients with OSA. **Materials and Methods:** ApneaLink™ is a single-channel screening device that measures nasal flow via a cannula connected to a pressure transducer to measure patient breathing. It automatically derives snoring and nasal flow and then derives apnea hypopnea index (AHI). We compared the AHI measured by ApneaLink device to that measured simultaneously by overnight polysomnography (PSG) in patients referred to the sleep disorders center with a clinical suspicion of OSA to assess the agreement between the two devices and determine specificity and sensitivity of ApneaLink in detecting OSA. The scorer of the PSG was blinded to the results of the ApneaLink. **Results:** Complete studies were obtained for 44 patients a mean age of 43.2 ± 11.7 yr and a body mass index of 33.1 ± 7.7 kg/m^2^. AHI was significantly lower in the ApneaLink 19.9 ±27.4 events/hr compared to PSG 35.2 ± 32.7 events/hr (*P*<0.001). Despite the fact that AHI in ApneaLink and PSG showed significant correlation (r=0.87, *P*<0.0001), Bland-Altman plot demonstrated a clear underestimation of AHI (especially hypopneas) by ApneaLink at all levels of OSA severity. Table 1 demonstrates the specificity and sensitivity of ApneaLink at different levels of OSA severity.

**Table 1:** Specificity and sensitivity of ApneaLink

AHI	Sensitivity	Specificity
>5	64.9	100.0
>10	58.1	92.3
>15	59.3	100.0
>20	66.7	100.0
>30	57.9	100.0

**Conclusions:** ApneaLink device is highly specific but not sensitive in detecting obstructive respiratory events at all levels of severity. Hence, using ApneaLink as the sole device for screening OSA may result in underestimation of the severity of OSA and missing patients with mild to moderate sleep disordered breathing.

## Critical care response team the most common trigger criteria associated with the highest ICU admission rate

**Essam N. Elkady, Alaa M. Gouda, Saad Al-Qahtani, Lian F. Fong, Yaseen Arabi**

King Abdulaziz Medical City, National Guard Hospital, Riyadh, Saudi Arabia

**Type:** Poster presentation

**Purpose:** To define the most common trigger criteria for critical care response team (CCRT) correlated with the highest ICU admission rate, this information will be helpful in guiding clinical decision for ICU admission and in developing scoring system to predict the need for ICU admission. **Materials and Methods:** Our hospital is 700 bed tertiary care center, RRT started November 2007, as 24/7 service lead by North American board certified intensivists. Cohort analysis of prospectively collected data of each RRT activation including reason of activation, final disposition of patient and patient's demographic data. We analyzed each factor separately as independent predictor of ICU admission within 24 hours, in 82 out of 283 patients the team was triggered for more than one reason however each factor was analyzed separately. **Results:** Total number of activation was 311 patients of which 26 patients were made DNR and remained on floor, 2 patients expired during activation, and the remaining 283 patients were analyzed.ICU admission rate was 50.5%, while 49.5% remained on floor. The most common activation criteria leading to ICU admission found to be decrease level of consciousness defined as drop of GCS 2 or more points from baseline (*P* 0.01) with 66.6% required ICU care, on contrary the most common criteria associated with non ICU admission was serious concern with no objective data, (*P*0.025) with 72% did not require ICU care. Most common trigger factor of RRT was due to tachypnea 71 patients of which 35 transferred to ICU, serious concern 63 of which 24 patients transferred to ICU, tachycardia 59 of which 31 transferred to ICU, Hypotension 57 of which 32 transferred to ICU, decrease level of consciousness 51 of which 34 transferred to ICU. **Conclusion:** Our data shows that Decrease level of consciousness has the highest association with the need for ICU admission compared to other factors and may be of higher score in any future scoring system predict the need of ICU admission.

## Open esophagomyotomy is better than Pneumatic ballon dilation in achalasia manegment: Long-time results

**Vahid Goharian, Amir Hosein Davarpanah Jazi, Sayyed Abbas Tabatabaee, Sayyed Mozafar Hashemi, Gholamreza Mohajery, Mojtaba Ahmadi Nejad, Mohsen Kolahdoozan**

Al Zahra Hospital, Isfahan University of Medical Sciences, Isfahan, Iran

**Background**: For many years different treatments have been used for achalasia. However, esophagomyotomy (ESM) and pneumatic balloon dilation (PBD) have been considered the treatments of choice. Despite new research, some controversies still exist. **Materials and Methods:** We compared patients who underwent open ESM (n = 19) with those who underwent PBD (n = 45). Data on age, gender, pre- and postprocedure weight, pre- and postprocedure radiologic findings, time of recurrence of clinical manifestations after management, type of surgery, and were collected via questionnaire. **Results:** There was significant difference in recovery of radiologic findings, weight gain and time of recurrence of clinical manifestations between the groups. Clinical rates of relapse for open ESM and PBD groups were 38.6% and 25%, respectively. **Discussion:** Considering the better long time outcomes of ESM to PBD, ESM is more reliable than PBD for management of achalasia.

**Key words:** Achalasia, pneumatic balloon dilation, surgical myotomy, radiologic findings, weight, time of recurrence of clinical manifestations

## The role of endobronchial ultrasound in assessment of mediastital lesions

**Tarek Safwat, Adel Khattab, Salwa EL Haddad, Yasser Mostafa, Emad Korraa, Ashraf Madkour, Wael Abd El Fattah**

Enlarged mediastinal lymph nodes and mediastinal masses are common diagnostic problem for chest physicians. In patients with lung cancer, evaluating mediastinal lymph nodes is crucial for proper staging and for assessing the extent of the disease. Endobronchial ultrasound (EBUS) is an imaging method that has been developed during the last decade, as a new diagnostic procedure for visualization of bronchial, peribronchial tumors and mediastinal lymph nodes. The aim of this study was to evaluate the role of EBUS in assessment of mediastinal lesions. Thirty patients suspected to have mediastinal lesions were enrolled in this study. They were subjected to; thorough history taking, clinical examination, chest X-ray, chest CT-scan, fiberoptic bronchoscopy, endobronchial ultrasound, EBUS-assisted TBNA and samples from suspicious sites were taken for cytological and/or histopathological examination. Results: showed that EBUS was able to detect mediastinal involvement in all the studied cases, while bronchoscopy suggested mediastinal involvement in only 14 of them (46.7%). Additionally, EBUS was superior to chest CT-scan and FOB in detecting 17 additive lymph nodes (constituting 24.3% of all detected lymph nodes) and also in detecting tumor invasion of the tracheobronchial wall in 6 patients out of 17 patients proved pathologically to have malignant mediastinal lesions (constituting 35.3% of studied malignant cases). EBUS-assisted TBNA had diagnostic yield of 83.3% (definitive cytological diagnosis had been reached in 25 out of 30 studied patients) and the majority of complications were mild, infrequent and the examination was never aborted. Conclusion: Endobronchial ultrasound provided valuable beneficial additional information beyond bronchoscopy and CT and EBUS-assisted TBNA is highly effective in diagnosing mediastinal lesions.

## Assessment of sleep pattern, energy expenditure and circadian pattern of body temperature for patients with acute coronary syndrome using the ArmBand: A preliminary report

**Al Otair H., Sharif M. M., BaHammam A.**

The University Sleep Disorders Center, College of Medicine, King Saud University

**Introduction:** Patients in acute care units have significant sleep disturbances, which may impact on their health. While many physiological parameters are monitored in patients in acute care units, there is no simple practical way to monitor sleep pattern in these patients. Therefore, we designed this study to assess the utility of a new portable device in assessing sleep pattern, energy expenditure and the circadian pattern of body temperature in patients in the coronary unit (CCU). **Methodology:** We studied the sleep pattern and circadian rhythm of consecutive patients admitted to CCU in King Khalid University Hospital, Riyadh, Saudi Arabia with the diagnosis of acute coronary syndrome (ACS). Patients on sedatives, mechanical ventilation and those with neurological disorders were excluded. We used the ArmBand (SenseWear Pro2 Armband, BodyMedia Inc, Pittsburgh, PA, USA) for data collection. The ArmBand is a validated metabolic body monitoring system that will record body temperature, total energy expenditure, sleep efficiency, and duration and the circadian distribution over 24 hours. The device was placed over the triceps during the first 24 hr in the CCU and upon transfer to the ward; the same measurements were recorded for a minimum of 24 hr. The collected data was downloaded into a PC and an advanced analysis was used to extract and analyze the needed information. **Results:** Seventeen patients with acute coronary syndrome (15 males) with a mean age of 57±7.8 yrs and of BMI 26 ± 4.8 kg/m^2^ were included in this preliminary report. They sleep for 6.7 ± 2.8 hr/24 hr divided into 3.7 ± 1.6 sessions. Energy expenditure was 1750.8 ± 203.6 kcal/day. Follow up monitoring was done for eight patients after transfer to the ward. Table 1 presents a comparison between the CCU and general ward stay. **Conclusion:** Arm Band is a promising tool for studying sleep pattern and metabolic changes in patients in acute care units. This preliminary report revealed that patients tend to take more daytime naps while in CCU. Once a good number of patients is collected, we will be able to study the circadian pattern of sleep and body temperature in patients in the acute care units.

**Table 1:** A comparison between CCU and general ward variables

Variable	CCU	Ward	*P* value
TST + Nap (min)	386.2 ± 197.5	386.6 ± 192.8	NS
TST (min)	284.4 ± 154.0	320.5 ± 146.2	NS
Night sleep sessions (from 21:00 – 06:00)	6.9 ± 5.0	4.38 ± 2.72	NS
Nap (min)	101.8 ± 90.4	66.1 ± 72.0	0.01
Daytime sleep sessions (Nap frequency)	4.4 ± 4.3	3.0 ± 3.9	NS
Total sleep sessions/24 hr	11.3 ± 7.6	7.4 ± 5.6	NS
Heat flex	66.8 ± 15.2	67.9 ± 11.5	NS
Skin temperature (°C)	32.4 ± 0.8	32.8 ± 0.5	0.037
Near body temperature (°C)	32.3 ± 0.7	32.7 ± 0.5	0.034
Energy expenditure kcal/min	1.2 ± 0.18	1.3 ± 0.1	0.09
METS	1.0 ± 0.1	1.1 ± 0.1	0.06

TST: Total sleep time at night (21:00-06:00).

## Mechanical ventilator adjustment in critical care unit: The current roles of clinicians

**H. Al-Otaibi, H. Maghraby, R. Mahajan, J. Hardman.**

Division of Anaesthesia and Intensive Care, University of Nottingham, Nottingham, UK

**Introduction:** The present study investigates the role of clinicians in context of mechanical ventilator (MV) adjustment. **Materials and Methods:** A prospective, observational, descriptive, non-interventional design conducted in two closed medical and surgical intensive care units at regional hospitals. Ventilator flow sheet and open-ended questionnaire were used to collect data. Clinicians were asked to record MV adjustment episodes and simultaneously complete data collection tools. **Results:** 187 ventilator adjustment episodes derived from 34 mechanically ventilated patients were evaluated. 94% of ventilator adjustments were physically made by respiratory therapists (RTs) whereas only 6% made by nurses. Decisions made to adjust MV were conducted by RT 138 (73%), medical staff 26 (14%), nurses 7 (4%), and in collaboration 16 (9%). Proportion of adjustments made by; RT: ventilator mode 20%, minute ventilation (V_E_) 16%, positive end-expiratory pressure (PEEP) 12%, pressure support (PSV) 13%, fraction of inspired oxygen (FIO_2_) 39%. Medical staff: mode 18%, V_E_ 36%, PEEP 3%, PSV 14%, FIO_2_ 29%. Collaborative adjustment: mode 23%, V_E_ 46%, PEEP 15%, FIO_2_ 16%. Nurses only adjusted PSV. **Conclusion:** The present data indicate that MV adjustments are shared amongst clinicians in critical care unit. However, RTs seem to assume more responsibility. Communications and collaboration between clinicians seem to be suboptimal.

## Effect of discontinuation of inhalation of corticosteroid on patients with COPD

**Hala Abdel-Hameed Mohamed, Mohamed El-Hosainy Magde, Mona hashem Allam**

Pulmonary and Critical Care Department, Faculty of Medicine, Minia University

**Background**: Studies of inhaled corticosteroids (ICS) in patients with irreversible airflow obstruction have provided conflicting results, some of them demonstrate no significant decrease in the rate of decline in forced expiratory volume in one second (FEV1) in other studies the mean FEV1 remains significantly higher in the steroid therapy group^14,15^. However, most of the studies that have evaluated these therapies have been short and powered to evaluate the impact on physiological end points, such as FEV1 values. Relatively few studies have evaluated the long-term effects of these interventions on clinical end points, such as healthrelated quality of life, COPD exacerbations, hospitalizations, or mortality. **Objectives:** To determine the effect of long term inhaled corticosteroids versus discontinuation of ICS treatment on lung function, exacerbations, and health status in patients with moderate to severe chronic obstructive pulmonary disease. **Patient and Methods:** Our study included 73 COPD male patients presented with moderate to severe COPD Every patient group are also classified into 2 subgroups according to the duration of inhaled steroids. **Results:** A significant improvement in FEV1 was observed in groups 1 (moderate obstructive) in continuation to use ICS for 6 months started with 56.60 ±4.70 to 63.80 ±10.74 after 3 ms. *P*<0.05 and to 68.10±11.58 after 6 ms *P*<0.01 also, FVC raised from 86.60±11.62 to 90.80±11.49 *P*<0.001 and to 97.30±12.97 *P*<0.05. There was a significant decrease in the number of exacerbations in the second 3months in patients with severe and very severe obstruction who continued on 6 ms. therapy of ICS. **Conclusions:** Inhaled corticosteroids alter the decline in FEV1 in patients with COPD, and significantly reduced exacerbations and the rate of decline in health status, while, the cessation of inhaled corticosteroid therapy in patients with severe COPD results in an increased risk of recurrent exacerbations.

**Key word*s:*** Inhaled steroids, chronic obstructed pulmonary disease, FEV1

## Relationship between skin prich test, peripheral eosinophilic count, serum total and specific IgE and severity of asthma in atopic asthma

**Hamdy A. Mohammadien, Hydi A. Mohamed^1^, Mohammad A. Elqady^2^**

^1^Departments of Chest and Clinical Pathology, ^2^Faculty of Medicine, Sohag University, Egypt

**Background:** Atopic sensitization is a well-known risk factor for asthma and bronchial hyperresponsivness. IgE-mediated responses contribute to allergy and asthma. Blood eosinophilis are known to be an indirect marker of airway inflammation in asthma. **Objective:** To evaluate the interrelationship between skin prick test, serum total and specific IgE levels, eosinophilia and asthma severity in atopic asthmatics. **Materials and Methods:** One hundried ten atopic asthmatics patients were randomly selected and 30 matched control subjects were included. All were submitted to detailed clinical history and examination, pulmonary function testing, skin prick testing to 17 common allergens, blood eosinophil counts and both total and specific IgE levels. Asthma severity was assessed according to GINA guidelines. **Results:** There was a highly significant relationship between total IgE, eosinophil counts and percent (*P*<0.0001) from one hand and severity of asthma from the other hand. There was a significant increase in severity of asthma with the increasing number of positive allergens in SPT and also when asthma was associated with other allergic diseases (*P*<0.04).Increased number of positive allergens was associated significant increase in the level of total IgE, eosinophil count and percent (*P*<0.0002). Total IgE was significantly higher in atopic asthmatics with eosinophilia than those without (*P*<0.0001). Eosinophil count and percent, total IgE and specific IgE were significantly higher in patients than controls (*P*<0.0001). The most common allergens for atopic asthma in our locality were D. pternoyssinus, followed by D.farinae then Cockroach extract. **Conclusions:** Atopic asthmatic patients with a higher serum concentration of both total IgE and specific IgE, blood eosinophilia and sensitivity to more than one allergens in SPT have a higher asthma severity.

**Key words:** Asthma, atopy, eosinophils, IgE, skin prich test

## Tracheobronchopathia osteochondroplastica: Presentation of ten cases and review of the literature

**Jabbardarjani Hamidreza**

Tracheobronchopathia osteochondroplastica (TO) is a rare benign disease of the endobronchial system with nonspecific symptoms and different treatment approaches. We report on a group of patients with TO and discuss their presentation and their treatment modalities. Between 2000 and 2006, the medical records of patients with TO were studied at the Interventional Pulmonary Unit of the Tracheal Disease Research Center at Masih Daneshvary Hospital, a referral center for respiratory diseases in Tehran, Iran. We analyzed and studied patients' demographics, symptom presentation, radiographic appearance, bronchoscopic findings, and their subsequent treatment. Of the 8760 patients who underwent flexible bronchoscopy (FOB) at our center over 6 years, 10 were diagnosed with TO; their median age was 51 years (range = 16–68 years) and 6 were men. Computerized tomography (CT) revealed mucosal irregularity and calcified nodules. Histopathologic examination of tissue biopsy through bronchoscopy was used for definitive diagnosis of TO. Bronchoscopy findings showed the endobronchial lesions to be firm and glossy. Cartilaginous nodules were present in central airways. Treatment included endobronchial Nd:YAG laser photoevaporation (LPE) (n = 10), coring of the lesions with the tip of the rigid bronchoscope (n = 4), and endobronchial stent placement (n = 1). Symptoms were considerably relieved in six cases but there was no significant improvement noted in three patients. One patient died after 6 years of treatment and follow-up. TO is a rare disease and the diagnosis should be suspected based on CT findings or bronchoscopic examination of the airways. Histopathologic examination is required for confirmation of diagnosis. Treatment is palliative and it includes LPE, coring through a rigid bronchoscope, and endobronchial stent placement.

## Central airway obstruction masquerading as difficult-to-treat asthma

**Jabbardarjani Hamidreza**

**Background and Objectives:** Large airway obstruction is well described as a cause of apparent asthma, unresponsive to the standard asthma therapy. Our purpose is to introduce multiple variants of other diseases that mimic asthma with failure to treat. **Materials and Methods:** Between the years 2005 and 2008, clinical data from 16 patients with difficult-to-treat asthma (from 3200 patients who were evaluated in our ward for any reason) were reviewed at Massih Daneshvary Hospital. **Result:** Of the 16 patients, 5 were men and 11 women. Mean age was 40.43 years (40.43±SD=16.49). Dyspnea was present in 93.75% of the patients. Chest X-ray was normal in 62.5% of patients. Computerized tomography scan was normal in 18.75% of the patients. Endobronchial lesion was found in 87.5% of patients in which 50% was benign and 50% malignant. Vocal cord dysfunction was found in 6.25% of the patients whereas 6.25% had external pressure on tracheal lumen. Mean duration of asthma treatment was 23.8 months (range, 3 to 96 mo). **Conclusions:** Asthma masqueraders include several conditions that may confound a correct diagnosis of asthma. A careful clinical examination, high index of suspicion, and appropriate analysis of spirometry are essential for the appropriate diagnosis and management of asthma. If there is no improvement after appropriate treatment, further evaluation should be performed.

**Key words:** Difficult-to-treat asthma, bronchoscopic evaluation

## Causes of pulmonary hypertension in down syndrome patients: The experience in a tertiary care center in Saudi Arabia

**Hanaa Banjar**

Department of Pediatrics, King Faisal Specialist Hospital and Research Center, Riyadh, Saudi Arabia

Children with Down syndrome (DS) have an increased risk for developing pulmonary hypertension due to multiple factors, including the presence of congenital Heart disease with persistent left-to-right shunts, chronic upper airway obstruction or abnormal pulmonary vasculature growth. In addition, patients with DS and congenital heart disease seem to develop pulmonary hypertension at a faster rate and have persistent disease after cardiac surgery compared to non-DS patients with similar defects. it was also found that DS patients have an increase incidence of PPHN compared to historical controls regardless of base line demographic. **Objectives:** To identify the possible contributing factors of pulmonary hypertension (PHT) in patients with Down syndrome, and identify the role of management intervention in improving PHT. **Materials and Methods:** Retrospective chart review for all Down syndrome patients that were referred to pulmonary services at KFSHRC-Riyadh with confirmed PHT by cardiac catheterization during the period 1998-2008. Demographic, clinical data, type of cardiac defect, use of vasodilator, diagnostic tests, morbidity, and mortality data were collected. **Results:** A total of 58 patients with Down syndrome were referred for recurrent respiratory symptoms, 36 (62%) patients had confirmed PHT by cardiac catheterization and or by Echo. Thirty five of 58 (60%) of patients are alive, 9 have died (16%), and 14 (24%) are lost follow up or referred to a local hospital. Thirty nine patients (68%) had cardiac defects: 14 (36%) as common A-V canal, 10 patients (26%) ASD, 7 patients (18%) VSD, and 8 (20%) had PDA. Five patients had combined ASD or VSD in addition to PDA, and the remaining 19 patients (32%) had no cardiac defects. Thirty four of 39 patients (87%) with cardiac defects, required cardiac surgery. Fifteen patients of 58 (26%) had signs and symptoms with obstructive sleep apnea (OSA) and 12 of them (21%) required adeno-tonsillectomy to improve their respiratory symptoms. Twenty one patients (36%) were treated for asthma symptoms. Another 13 patients (22%) had radiological signs of gastroesophageal reflux (GER), 6 of them required Nissen fundoplication. Twelve patients (21%) required home oxygen. Five of 34 patients (15%) who had repair of their cardiac defects, continued to develop PHT, 2 of them improved on Sildenafil. Three patients from the group that did not have repair of their cardiac defect due to the severity of the PHT were treated with vasodilator, 2 of them improved on Bosentan form functional class III to functional class 1 and the third one improved on Sildenafil.

**Conclusion**: Pulmonary hypertension is common in Down's syndrome patients with or without cardiac defects. Physician should be aware of other factors that may cause PHT such as: OSA, asthma and GER and should screen for them routinely in such patients.

## Assessment effect of using nutritional guideline on spirometric tests in patients with COPD

**Mansooreh Tajvidi, Mandana Arash, Mitra Parsinia**

Karaj Islamic Azad University

**Introduction:** Chronic obstructive pulmonary disease (COPD) is the fourth leading cause of death and its prevalence is increasing. Nutritional depletion is a common problem in COPD patients.This problem adversely affects morbidity and mortality. Aim of the present study is the assessment effect of using nutritional guideline on spirometric tests (FEV1, FVC, FEV1/FVC) in patients with COPD. **Materials and Methods**: This research is the quasiexperimental study. Random sampling on 30 COPD patients has been done. In the first stage hospitaling patients filled demographic questionnaires and spirometric tests were measured. Then nutritional guideline was educated face to face in 4 sessions of 15 minutes. In the second stage spirometric tests were measured after 1 month and 3 months. The researcher followed up samples in these periods by calling them. Finally spirometric tests (FEV1, FVC, FEV1/FVC) were compared before using nutritional guideline and after it. Data were analyzed by SPSS. **Results**: 30 patients with COPD entered the study (16males and 14 females). Significant differences were seen between mean FEV1 before intervention (50.9%) and 1 month after intervention (66.49%) (*P*=0.004) and 3 months after intervention (87.75%) (*P*=0.007). There were significant differences between mean FEV1 after 1month (62.8%) and 3 months (83.9%) (*P*=0.04). There were significant differences between mean FVC before intervention (46.26%) and 1 month after intervention (65.23%) (*P*=0.01) and 3 months after intervention (81.88%) (*P*=0.01). **Conclusion**: Our results suggest that patients with COPD must be inform about factors (such as some nutritional factors) that could influence in their respiratory conditions. they must be guided what foods cause their respiration to be worse or better.

## Left ventricular hypertrophy in adolescents with obstructive sleep apnea: Egyptian experience

**Zaghloul M. A., Khattab G., Al-badawy S.^1^, Barakat M. M.^2^, Khalifah M. A.^2^**

Al-azher University Hospitals, ^1^Cairo University Hospitals, Cairo, and ^2^King Fahd Hospital, Jeddah, KSA

**Context:** Obstructive sleep apnea (OSA) has been shown to be an independent risk factor for cardiovascular disease in adults. However, there are limitations in the extent to which the cardiovascular sequalae of OSA are being studied in adolescents. **Patients and Methods:** To investigate the cardiac changes in adolescents with OSA, echocardiographic right and left ventricular (RV, LV) dimensions and LV mass index were measured in 94 adolescents, 56 adolescents with OSA and 38 adolescents with primary snoring (PS). The study was conducted at Al-zaher university hospitals, and Cairo university hospitals, Cairo. **Results:** Ninety four adolescents completed the study, which showed that LV mass index and relative wall thickness were greater in the OSA group compared with those with PS (*P* = 0.012 {0.0001}), respectively). An apnea–hypo-apnea index of more than 10 per hour was significantly associated with RV dimension above the 95th percentile (odds ratios, 6.9; 95% confidence interval, 1.7–32.3) and LV mass index above the 95th percentile (odds ratios, 11.5; confidence interval, 1.7–64.3). **Conclusion:** Abnormality of LV geometry was present in 15% of children with PS compared with 40% of children with OSA. We conclude that OSA in children is associated with increased LV mass.

**Key words:** Obstructive sleep apnea, adolescents, left ventricular hypertrophy, cardiovascular abnormality

## Diagnostic evaluation of patients with chronic non-productive cough: Saudi experience

**Zaghloul M. A., Barakat M. M., Khalifah M. A., Khattab G.**

King Fahd Hospital, Jeddah, KSA

**Context:** Bronchial Asthma (BA), post-nasal discharge syndrome (PNDS), and gastro-esophageal reflux disease (GERD) account for many cases of chronic non-productive cough. Each may contribute to cough even when silent clinically, and failure to identify their contribution may lead to treatment failure. **Patients and Methods:** Eighty six patients (non-smokers with normal chest radiographs and spirometry measurements) referred with chronic non-productive cough persisting for more than three weeks as their only respiratory symptom underwent histamine challenge, home peak flow measurements, ear, nose and throat (ENT) examination, sinus CT scanning, and 24 hour esophageal pH monitoring. Treatment was prescribed on the basis of diagnosis informed by results of investigation. **Results:** Eighty six patients (56 males) of mean age 45.5 years (range 20-70) and mean cough duration 62 months (range 4-120) were evaluated. On the basis of a successful response to treatment, a cause for the cough was identified in 70 patients (82%) as follows: Bronchial Asthma (BA) (20 cases), post-nasal discharge syndrome (PNDS) (18 cases), gastro-esophageal reflux disease GERD (16 cases), and more than one etiology (16cases). Histamine challenge correctly predicted Bronchial Asthma (BA) in 30 of 34 (88%) positive tests. Sinus CT scan and ENT examination each had low positive predictive values for PNDS, 24 of 36 (67%) and 20 of 32 (63%) positive cases respectively). Negative, histamine challenge and 24 hour esophageal pH monitoring effectively ruled out BA and GERD respectively, as a cause for cough. **Conclusion:** This diagnostic protocol helps the accurate direction of treatment, while BA, GERD and PNDS remain the most important causes of chronic non-productive cough, but still we should consider, a group with un-identifiable etiology remains. Sinus CT scan and ENT examination each had low positive predictive values for PNDS, 24 of 36 (67%) and 20 of 32 (63%) positive cases respectively, suggesting that upper airways disease frequently co-exists but does not always contribute to cough.

**Key words:** Chronic non-productive cough, diagnosis, treatment

## Obesity and sleep duration in young Saudi adults: Is there an association?

**Al Fotaih M., Basuliaman B., Alfakhri A., Sharif M. M., BaHammam A.**

The University Sleep Disorders Center, College of Medicine, King Saud University

**Introduction:** Many studies showed a rapid increase in the prevalence of obesity in Saudi Arabia. Some epidemiological studies have linked obesity to short sleep duration. While laboratory studies have linked short sleep duration to hormonal changes that enhance obesity. The link between sleep duration and obesity has not been assessed in our geographical area before. In view of the fact that a number of studies showed that Saudis tend to have short sleep duration due to late bedtime, we conducted this study to assess the association between sleep duration and other habits on one side and weight on the other side in young Saudis. **Materials and Methods:** Using anonymous questionnaires, data were collected from 1025 university students (407 females and 552 males) aged 17 to 30 years (mean age was 21) by simple random manner. The response rate was 76%. The outcome measure was obesity, defined as a body mass index ≥30 kg/m^2^. The following data was collected; age, sex, smoking, self-reported weight and height, physical activity and self-reported sleep duration. Data was collected by trained medical students. **Results:** The estimated prevalence of obesity was 18% for men and 5% for women (the overall percentage was 13%). Most of the obese people slept between 6 to 8 hours (20% slept less than 6 hours, 60% slept between 6 and 8 hours, 19.6% slept more than 8 hours). Although there was negative correlation between sleep duration and obesity (r=-0.49 and *P*>0.05). Logistic regression analysis revealed that obesity was associated only with smoking, sport activity and gender difference. People who smoked showed a higher odd ratio for obesity, OR=2.0 [95% confidence interval, 1.2-3.44] than those who don't smoke. Eighty five percent of obese people reported to don't have a regular sport activity. The male gender tend to have a higher BMI then the females( 22% of males), OR=4 [95% confidence interval, 2.22-6.75]. In addition, the prevalence of obesity was lowest in single people, physically active people and those who took regular naps. **Conclusion:** This study did not show association between sleep duration and obesity. Obesity was associated with smoking, male gender and sedentary life style.

## Prevalence of symptoms and risk of sleep apnea in a sample of middle-aged Saudi females using the berlin questionnaire

**Mohammad Alrajeh, Maria Arafah, Fatimah Al-ibrahim, Ahmed BaHammam**

The University Sleep Disorders Center, College of Medicine, King Saud University

**Introduction:** Obstructive sleep apnea (OSA) is a common sleep related breathing disorder associated with increased morbidity and mortality. No previous studies have estimated the prevalence of OSA in Saudi females. We conducted a previous study to identify the percentage of Saudi middle-aged males at risk of developing OSA. Now, we conducted another study to identify the percentage of Saudi middle aged females at risk of developing OSA. **Materials and Methods:** We administered the Berlin Questionnaire (which is a pre-designed, validated, standardized questionnaire that includes questions about snoring, witnessed apneas, self-reported hypertension, and daytime sleepiness and was found to predict an RDI of >5/hr with a sensitivity of 0.86, a specificity of 0.77, a positive predictive value of 0.89, and a likelihood ratio of 3.79) to Saudi middle-aged females (a mean age of 43.7±6.3 yr), attending the primary health care center (PHCC) in (KKUH) in Riyadh. Based on the questionnaire, individuals were classified into high risk and low risk groups for OSA. This questionnaire was administered to consecutive patients attending the PHCC. Data was collected by trained medical students. **Results:** Four hundred subjects were surveyed with a BMI of BMI 30.3±7.2. Among the study group, 40.8% reported snoring (everyday in 15%, 3-4 times a week in 7.5%, and 1-2 times a week in 9.8%). Twenty six percent of the recipients had breathing pauses while asleep (6.2% almost everyday, 4% 3-4 times a week, 5% 1-2 times a week, 10.8% 1-2 a month). Thirty two percent reported fallen asleep while in the car. From these recipients, who answered the questionnaire, 24.8% were known to be hypertensive. Based on the Berlin questionnaire stratification for risk of OSA, 45.25% were considered as high risk patients for OSA. **Conclusion:** In primary care setting, the prevalence of symptoms of OSA among middle-aged Saudi ladies is very high. Almost, one of every two middle-aged Saudi ladies is at risk of OSA and benefit from proper evaluation for OSA.

**Table:** 

	Our study (n=400) F	BaHammam *et al.* (n=556) M	Netzer *et al.* (n=744) M + F	Heistand *et al.* (n=1506) M + F	Sharma *et al.* (n=180) 80% males
Mean age	43.7±6.3	45.2 ± 9.3	48.9 ± 17.5	49	-
Snoring	40.8%	52.3%	52.2%	59%	-
Day time fatigue >3 time a week %	40.2	19.2	38.8	26	-
Drowsy driving %	32	30	19.9	32	-
HTN (known)%	24.8	18	26	29	53
High risk%	45.25	33.3	37	Males 31 Females 21	44

## Study of tuberculous infection rate and characteristics of tuberculosis patients in a centeral province of Iran

**Mohammad Hossein Salari, Abbas Mirshafiey, Mohammad Bagher Khalili, Ardeshir Behmardi Kalanteri, Gholamreza Hassanpour, Ali Asghar Sadrabadi**

Department of Pathobiology, School of Public Health and Institute of Public Health Research, Tehran University of Medical Sciences, Iran

**Objective:** Tuberculosis is a continuous threat for health in all parts of the world. An estimated one-third of the world's population is infected with the *Mycobacterium tuberculosis* and 7 to 8 million people develop TB disease each year. The purpose of this study was to investigate of tuberculous infection rate and characteristics of tuberculosis patients in a Centeral province of Iran. **Materials and Methods:** During the study period, 3885 suspected tuberculosis patients (1820 males and 2065 females; aged 8-85 years) who had been referred to the Yazd referral polyclinic were investigated by Ziehl Neelsen staining and culture method and questionnaire was completed for each subject. Then, collected data were analyzed by statistical package for social science (SPSS) and chi-square program. **Results:** The results show that, of the total suspected tuberculosis, 604 cases were found to be positive for tuberculosis. The average annual rate of tuberculosis was 26.8 cases per 100000 population (23.1/100000 males and 31/100000 females). Results of statistical analysis test show significant differences between sexes (df=1, χ^2^=4.64, *P*<0.05) and also age groups of patients (df=5, χ^2^ =301.16, *P*<0.001). The number of pulmonary and extra-pulmonary tuberculosis was 458 cases (61/100000) and 146 cases (19.4/100000), respectively. Our findings show that, the number of cured was 403 cases (66.7%), transferred to a different catchment area 51 cases (8.4%), treatment failure 107 cases (17.7%) and deaths 43 cases (7.1%). Out of total tuberculosis patients, 382 cases (63.2%) were Iranian, 218 Afghanian refugees (36.1%) and 4 cases (0.7%) had other nationalities. **Conclusion:** It seems that, despite the efforts, which have been done for prevention, diagnosis and treatment of patients with tuberculosis, it is still considered as a threat for health in the Yazd province, Iran.

**Key words:** Rate, tuberculosis, characteristics, patients, centeral province, Iran

## Prevalence and characteristics of obesity hypoventilation syndrome in patients with obstructive sleep apnea

**Mohammed Alharbi, Munir Chaudhry, Shaffi Shaikh, Ahmed BaHammam**

The University Sleep Disorders Center, College of Medicine, King Saud University

**Introduction:** No study has addressed obesity hypoventilation syndrome (OHS) in Saudis. This study was conducted to assess the prevalence and characteristics of OHS in patients referred to the sleep disorders center with obstructive sleep apnea (OSA). **Materials and Methods:** Retrospectively, we reviewed the records of 763 patients who underwent polysomnography in the University Sleep Disorder Centre in King Khalid University Hospital and diagnosed to have OSA. OSA and OHS diagnoses were considered according to ICSD criteria. Demographics, polysomnographic parameters, arterial blood gases, pulmonary function test (PFT) and baseline characteristics were explored. **Results:** The whole group age was 47.5 ± 13.3 yr and BMI 36.1 ± 9.5. OHS was identified in 64 of 763 OSA patients (8.4%) and the rest had pure OSA. OHS patients were older, had lower FEV1 and FVC, higher AHI and higher BMI than pure OSA patients. Awake PaCO_2_ positively correlated with BMI and negatively with the mean nocturnal oxygen saturation. **Conclusion:** The prevalence of OHS among Saudi OSA patients is comparable to that reported in the literature. Compared to pure OSA, OHS patients had more co-morbidities, were older and demonstrated higher BMI and AHI.

## What happens during REM sleep in patients with shrinking lung syndrome?

**Chaudhary M., Alhrabi A., BaHammam A.**

The University Sleep Disorders Center, College of Medicine, King Saud University

The shrinking lung syndrome (SLS) is a rare manifestation of systemic lupus erythmatosis (SLE). It is characterized by unexplained dyspnea, a restrictive pattern on pulmonary function tests and elevated hemi-diaphragms. There are no reports in the literature describing the respiratory changes during sleep in this group of patients. As the diaphragm is not functioning well in these patients, we hypothesize that patients with shrinking lung syndrome might not be able to sustain Rapid Eye Movements (REM) sleep and in case they progress into REM might have significant desaturation during REM sleep as other muscles will be paralyzed, which may result in significant hypoventilation. This abstract reports a case of a 25 years old Eritrean female with a BMI 26.75 known to have SLE, presented with a three month history of progressive shortness of breath. She was tachypnoic with a respiratory rate of 26/minute and a normal physical examination otherwise. Radiological studies revealed elevated hemi-diaphragms and absence of lung parenchymal or pulmonary vasculature pathology. Flouroscopy revealed a low volume thorax with significantly decreased yet symmetrical movements of both hemi-diaphragms. Overnight sleep study showed that the patient progressed normally into REM sleep which constitutes 19% of total sleep time. During REM, the patient has mild obstructive hypopneas (AHI=11/hr) that resulted in minimal desaturation (SaO_2_>90%). The fact that this patient had a normal quantity of REM sleep with minimal desaturation suggests that this group of patients may achieve inspiratory recruitment of extradiaphragmatic muscles in both tonic and phasic REM, which suggests brainstem reorganization as occurs in patients with chronic diaphragm paralysis. Therefore, the sleep study montage should include EMG monitoring of accessory respiratory muscles like sternocleidomastoids to document lack of hypotonia of accessory respiratory muscles during sleep.

## Congenital central hypoventilation with PHOX2B mutation in a Saudi child: A case report and review the literature

**Muslim Mohammed alSaadi**

The University Sleep Disorders Center, Pediatric Departments, College of Medicine, King Saud University, Riyadh, Saudi Arabia

Congenital central hypoventilation syndrome (CCHS) is a rare disease that is characterized by failure in the autonomic control of breathing. Recent reports have identified mutation of the paired mesoderm homeobox protein 2b (PHOX2B) gene as playing a major role in CCHS. Increasing polyalanine repeat number is associated with a more severe clinical phenotype. We report a four year old Saudi boy with the clinical manifestations of apnea and cyanosis required immediate mechanical ventilation at age of 5 minutes of life. Throughout the hospital course, he shows frequent hypoventilation with hypercapnia and hypoxemia occurred during sleep after weaning from the ventilator. Physical examination was normal for age while awake. Laboratory evaluation revealed that chest radiograph, electrocardiogram, echocardiogram, and fluoroscopy of the diaphragm were all normal. MRI showed no evidence of brainstem abnormalities. Screening studies for inborn errors of metabolism were negative. The patient was referred to the university sleep laboratory at the age of 6 months as failure to wean from mechanical ventilation for sleep study and clinical confirmation of the diagnosis of CCHS, which was confirmed polysmonographically and by molecular genetics study. He was discharged with life long home ventilator support via tracheostomy. This is the first case report demonstrating congenital central hypoventilation syndrome due to PHOX2B mutations in Saudi population.

## An audit of chest tube thoracostomy, Shaab Teaching Hospital, 2007

**O. E. Y. Elhag, A. M. Zaki^1^, M. Mirgani^2^**

Shaab Teaching Hospital, Khartoum, Sudan, ^1^Sudan Medical Specialization Board, ^2^Faculty of Medicine, University of Khartoum

Use of tube thoracostomy in respiratory care units for evacuation of air or fluid from the pleural space has become common-place, although considered a simple procedure, complication rates have been reported to be 2-25%. **Objective:** To determine the demographic pattern, indications and complications associated with tube thoracostomy at Shaab Teaching Hospital (STH) Khartoum-Sudan. **Materials and Methods:** A prospective case series of all patients who underwent tube thoracostomy during a 12 month period (January to December 2007) at Shaab teaching hospital were studied, Demographic data, clinical features, duration of drainage, complications and outcomes were analyzed. **Results:** A total of 77 cases were studied, male: female ratio was 1, 6:1. (50, 6) were smoker, thirty four (44.2%) were over 45 years of age. Infective cases accounted for 71.4% (55) of the cases that had tube thoracostomy. Eighteen patients (23.4%) had tube drainage for 10 days or less. Complication rate was 42,9% (33), mostly mild, Failure rate of 11.7% (9) was recorded for the procedure. A mortality of 1.3 (1) was recorded but there was no procedure related death. Eleven patients (14.3%) required further surgery. **Conclusion:** Tube thoracostomy is a simple and efficacious procedure for the treatment of pleural diseases. A larger study to confirm or refute these findings must be performed before any change in established safe practice.

## A study on prevalence of drug resistance in drug defaulter pulmonary tuberculosis

**O. A. Khair^1^, O. E. Abdali^2^, M. F. Kamal^1^, A. M. Abdelsalam^3^, N. Y. Ibrahim^3^, A. M. Zaki^2^**

^1^Department of Respiratory Medicine, City Hospital, Dudley Road, Birmingham, ^2^Sudan Medical Board of specilisation, Khartoum, Sudan, ^3^National TB reference Laboratory, Khartoum, Sudan

**Background:** The emergence of resistance to drugs used to treat tuberculosis (TB), and particularly multidrug-resistant TB (MDR-TB), has become a significant public health problem in a number of countries and an obstacle to effective global TB control. **Materials and Methods:** This is a prospective cross sectional study to estimate the magnitude of MDR tuberculosis in 2 Hospitals in Khartoum, Sudan. 111 patients who had defaulted their tuberculosis treatment on previous occasions and had presented to the hospital with several symptoms were studied. All patients provided sputum, which was examined for the presence of acid fast bacilli (AFB) by Ziehl-Neelsen stain. Sputa were also sent to the reference laboratory for mycobacterial culture and drug susceptibility testing. All culture positive sputa had drug sensitivity tested to the first line anti-TB drugs used in Sudan namely Streptomycin, Isoniazid, Rifampicin and Ethambutol. **Results:** Out of the 111 patients, 29.7% (n=33) were AFB sputum smear positive and 40.5% (n=45) were sputum culture positive for mycobacterium. Sensitivity testing revealed that 48.9% (n=22) were resistant to Streptomycin, 62.2% (n=28) were resistant to Isoniazid, 55.6% (n=25) were resistant to Rifampicin and 37.8% (n=17) were resistant to Ethambutol. 42% ( n=19) of the patients were resistant to Rifampicin and Isoniazid only, while 26.6% (n=12) were resistant to all the first line drugs (Streptomycin, Isoniazid, Rifampicin and Ethambutol). **Conclusion:** This study showed that the prevalence of MDR tuberculosis among the defaulters in Khartoum is much higher than what was reported previously. This study highlights the extent of the problem of drug resistance in Khartoum and emphasises the need for proper treatment and strengthening of the short course direct observed therapy strategy.

**Key words:** TB, MDR (multidrug resistance tuberculosis), Sudan

## The clinical efficacy, patient adherence to treatment and side effects of two 6 months regimes for the treatment of pulmonary TB

**O. E. Abdali, O. A. Khair^1^, M. F. Kamal^1^, A. M. Abdelsalam^2^, N. Y. Ibrahim^2^, H. K. ElKheir, Zaki A. Mahjoob**

Sudan Medical Board of Specilisation, Khartoum, Sudan, ^1^Department of Respiratory Medicine, City Hospital, Dudley Road, Birmingham, ^2^National TB reference Laboratory, Khartoum, Sudan

Tuberculosis remains a major public-health problem in Sudan, despite national efforts to improve case identification and treatment compliance. **Objectives:** To evaluate the clinical efficacy, patient adherence to treatment of two 6 months regimes for the treatment of pulmonary TB in Khartoum. **Design:** Randomized prospective hospital based controlled study. **Setting:** The tropical Hospital Abu Anga in Omdurman. **Study Population:** All TB cases reported between September 2001 to September 3003. **Outcome Measure:** The proportion of TB cases completed treatment and cured and the number of patients who discontinued treatment. **Results:** A total of 823 patients with sputum positive pulmonary tuberculosis were enrolled (Male: 518 (63%) and Female: 305 (37%). Patients with renal, hepatic, diabetic, cardiac problem and pregnancy were excluded from the study. Patients were randomly selected into two groups (A and B). group A was given Streptomycin, isoniazid, rifampicine, and pyrazinamide. group B were given same regime except that ethambutol was given instead of the streptomycin. All patients received 4 drugs in the intensive phase and 2 drugs (rifampicine and isoniazid) in the continuation phase. 60% of patients were cured after 6 months of treatment in group A compared to 83% in group B. 16% and 20% of patients discontinued treatment in group A in the intensive phase and the continuation phase respectively, compared to 3% and 1% of patients in group B respectively. **Conclusions:** Both treatment regimes are effective in the treatment of patients with TB. Treatment regime containing streptomycin is associated with a lower rate of cure and higher rate of treatment discontinuation.

**Key words:** TB, MDR (multidrug resistance tuberculosis), Sudan

## Event related evoked potentials in chronic respiratory encephalopathy

**R. Zaidan, A. R. Al Tahan, S. Jones^1^, A. Husain^2^, A Mobeireek, A. Bahammam**

Department of Medicine and ^2^Department of Physiology, College of Medicine, King Saud University, Riyadh, Saudi Arabia, ^1^Department of Neurophysiology, Institute of Neurology, London, U.K

**Background:** Cognitive event-related potential (P300) is an index of cognitive processing time. It was found to be prolonged in dementia, renal and hepatic encephalopathies, but was not extensively assessed in respiratory failure. **Objective:** To evaluate P300 changes in patients with respiratory failure, and especially those with mild or subclinical hypoxic-hypercapnic encephalopathy. **Materials and Methods:** Auditory-event related evoked potential P300 latency was measured using oddball paradigm, in patients with respiratory failure due to any cause (PO_2_ should be 75 mm/Hg or less). Apart from blood gases measurement, patients underwent mini-mental state examination. Patients' performance was compared with that of a matched normal control. They were admitted into the study from out-patient clinics and wards at King Khalid University, and Sahara Hospitals. **Results:** 34 patients (12 women and 22 men) were admitted to the study. Ages range from 19-67 years, with a mean of 46.1 years. Respiratory failure was severe or very severe in 11 patients (33%), and mild or moderate in the rest (66%). Mean values for PO_2_ and PCO_2_ were 63.7 and 45.2 mm/Hg respectively. PH mean was 7.4 and O_2_ saturation 90.7%. P300 latency ranged from 218 to 393 millisecond, with a mean of 338.4 millisecond. In comparison with that of control (309.9 milliseconds), there was significant difference (*P*=0.007). P 300 amplitude differences were however not significant. No significant difference in MMSE noted between the mild and sever respiratory failure. Results of detailed neuro-psychological assessment were clearly abnormal but limited by the small number of tested patients. P300 latency changes correlated significantly with age, as well as severity of respiratory failure. P300 was also significantly delayed whether hypoxia occurred with or without hypercapnia. **Conclusion:** Results shows significant delay of P300 latency in patients with severe and mild respiratory failure. This was associated with sub-clinical encephalopathy in most patients, evidenced by near normal MMSE score.

## The use of thoracoscopy to improve medical students' interest and understanding of thoracic anatomy

**S. Alnassar, J. Clifton, R. J. Finley, R. Sidhu**

Department of Surgery, University of British Columbia, Vancouver, BC

**Objective:** The evolution of the Minimally Invasive Surgery (MIS) has a great potential for improving both health care and medical education. MIS thoracoscopy, for example, can be a useful teaching tool and offers a link between clinical medicine and basic science. In this pilot study we developed a video-based educational tool designed for learning thoracic anatomy. Our objective was to examine whether integrating this tool into medical school anatomy training, would increase students', stimulation and motivation for learning anatomy. **Materials and Methods:** A video-based educational tool for thoracic anatomy was developed by recording different thoracoscopic procedures, focusing on intraoperative live thoracic anatomy. The tool was then incorporated into a pre-existing integrated program for first year medical students (n=150). The tool included cadaver dissection of the thorax and review of clinical problem scenarios of the respiratory system. Students were guided through a viewing of the videotape of thoracoscopic procedures which demonstrated live anatomy of the thorax (15 min). At the end of the presentation, medical students were asked to complete a 5-point Likert type questionnaire assessing he video and its usefulness. **Results:** The questionnaires were completed by 119 medical students. Most students were satisfied with the thoracoscpic video presentation as a teaching tool (mean score 4.39 ± 0.65) and thought that it increased their interest and understanding of thoracic anatomy (mean score 4.63 ±0.58, 4.10 ± 0.89). The majority would like to see this new teaching tool implemented into the anatomy curriculum (mean score 4.66 ± 0.66). The thoracoscopic video presentation also increased students interest in surgery as future career (mean score 4.19 ± 0.83). **Conclusion:** Incorporating live surgery via thoracoscopic video presentation in the gross anatomy teaching curriculum had high acceptance and satisfaction scores from first year medical students. The video increased students interest in learning, in clinically applying anatomic fact, and in surgery as a future career. Further studies are needed to evaluate the objective gain in knowledge associated with this teaching tool before implementing it in undergraduate medical curriculums.

## Reference values of exhaled nitric in healthy adults: Inverse correlation with body mass index v

**Syed S. Habib, Abdullah A. Abba, Mohammed Al Zoghaibi, Mohammed M. F. S. Beg**

Department of Physiology, College of Medicine and King Khalid University Hospital, King Saud University, Riyadh, Saudi Arabia

**Introduction:** Measurement of fraction of exhaled nitric oxide (FE_NO_) is widely used as a marker of inflammation in the airways. Values are however dependent upon a number of factors including gender, race and anthropometric characteristics. There is a lack of data on the normal values of FE_NO_ in the general adult Saudi population. The objective of the study was to determine the reference values of FE_NO_ among healthy, non-smoking male Saudi population and to determine if there is any correlation of FE_NO_ with age, height, weight and body mass index. **Materials and Methods:** One hundred and twenty one healthy, non-smoking, non-atopic, adult male Saudi subjects were studied. All subjects underwent spirometry. Exhaled nitric oxide was measured online by chemiluminescence procedure with single-breath technique using American Thoracic Society guidelines. **Results:** The salient results in the study are that the FE_NO_ measured at expiratory flow rates of 50 mL/s ranged between 7.66 parts per billion (ppb) and 46.6 ppb (mean 22.79 ± 8.13), with >84% of subjects recording levels less than 30 ppb and >95% less than 40 ppb. There was significant negative correlation between FE_NO_ and weight (r = -0.3888, *P*< 0.05) and BMI (r = -0.238, *P* < 0.05). **Conclusion:** The reference values of FE_NO_ for non-smoking, non-atopic male Saudi patients fall between 7.66 ppb and 46.6 ppb (mean 22.79 ± 8.13). A negative correlation was observed between FE_NO_ and BMI and body weight in this normal population. Further studies are needed to define the figures for females in this country.

## Chronobiology in young Saudis

**Shaya Alshaya, Wael Almestehi, Abdurrahman Albatli, Ahmed BaHammam**

The University Sleep Disorders Center, College of Medicine, King Saud University

**Objectives:** The circadian rhythm (chronobiology) has not been assessed in young Saudis before. Therefore, we conducted this survey to assess chronobiology in young Saudi males. **Materials and Methods:** Participants in this cross sectional study were healthy students studying at different colleges at King Saud University, Riyadh, Saudi Arabia. The study was conducted during February 2008. Around 1000 copies of a self-administered questionnaire were distributed to the participants in order to determine their demographics, sleeping patterns and habits. Chronobiology was assessed using a validated questionnaire (Morningness-Eveningness Questionnaire (MEQ)), which divides the circadian pattern into definitely morning (DM-type), moderately morning (MM-type), definitely evening (DE-type), moderately evening (ME-type) and neither type (N-type). **Results:** The final analysis included 759 students. The mean age is 20.70 ± 1.77 yr. Four hundred and seventeen participants (54.9 %) were defined to be of N-type, 175 (23.1%) as ME-type, 131 (17.3 %) as MM-type, 29 of (3.8 %) as DE-type, and 7 (0.9 %) as DM-type. **Conclusion:** The study revealed that young Saudis have higher percentage of the eveningness measures compared with other published studies (see the table below). This means that young Saudi prefer to do most of their activities in nighttime and sleep during daytime.

**Table:** 

	DM	MM	N	ME	DE
Current study	3.8	23.1	54.9	17.3	.9
Non Saudi (Goldman *et al*)	1.5	21.3	51.2	18.1	7.8

R Goldman, P Bruss, D Mitchell. The 73rd annual meeting of the Midwestern Psychological Association, Chicago, May 2001.

## Predictive factors of anastomotic stricture after transhiatal esophagectomy

**Vahid Goharian, Amir Hosein Davarpanah Jazi, Sayyed Abbas Tabatabaee, Sayyed Mozafar Hashemi, Gholamreza Mohajery, Mojtaba Ahmadi Nejad, Mohsen Kolahdoozan**

Al Zahra Hospital, Isfahan University of Medical Sciences, Isfahan, Iran

**Background:** By increased experience of surgeons, the mortality rate of Transhiatal esophagectomy (THE) is markedly reduced, so quality of life is more important issue after operation. The aim of this study was to determine the incidence of anastomotic stricture and predictive factors contributed to this complication after THE. **Materials and Methods:** We present a prospective study carried out in Al Zahra hospital between 61 patients with esophageal cancer from 2000 to 2007. The data of anastomotic leak, respiratory complication, reoperation, other complication, operation time, intraoperative bleeding and anastomotic stenos is evaluated. The data completely registered and analyzed by SPSS ver.13. **Results:** Leak of anastomosis, respiratory complication, reoperation, and overall complication were significantly correlated with anastomotic stenosis. **Conclusion:** For reducing the incidence of anastomosis stricture after THE improvement of respiratory condition as well as decreasing risk factors of anastomosis leak must be considered.

**Key words:** Anastomotic stricture, transhiatal esophagectomy, anastomosis leak, respiratory complication, anastomosis leak

